# Network Structure Predicts Changes in Perception Accuracy of Social Relationships

**DOI:** 10.3389/fpsyg.2018.02348

**Published:** 2018-11-26

**Authors:** João R. Daniel, Rita R. Silva, António J. Santos

**Affiliations:** ^1^William James Center for Research, ISPA – Instituto Universitário, Lisbon, Portugal; ^2^Social Cognition Center Cologne, University of Cologne, Cologne, Germany

**Keywords:** perception accuracy, affiliative networks, transitivity, elementary school children, young adolescents

## Abstract

The goal of this study was to test how changes in perception accuracy of affiliative networks (i.e., the ability to accurately identify who affiliates with whom) are related to an important structural feature of peer groups- the likelihood of children to affiliate with mutual partners (transitivity). Data from three longitudinal samples (two from elementary school children and one from young adolescents; *N* = 257, 618 observations) show that children and adolescents in classrooms with a higher proportion of transitive relationships are better at perceiving who affiliates with whom, and that increases in transitivity associate with increases in perception accuracy. This is the first study to show that structural features of peer groups relate with individual perceptions of affiliative relationships, providing further evidence that these features have an important role in promoting individual adaptation and supporting previous suggestions that classroom-variables play a role in fostering accurate perceptions of social relationships.

## Introduction

In any given classroom there are a number of peers with whom a given child can interact and they are fundamental for the socialization of beliefs, attitudes, values, and behaviors. Despite a wide range of available partners, social interactions are not random. From early on, specific preferences emerge and with time peer relationships become more complex and structured. The existence of transitive relationships within peer groups is likely the main structural feature that differentiates a social network of positive interpersonal ties from a network of random ties (Davis, 1970). In colloquial terms transitivity refers to the likelihood of two people with a common friend being friends themselves. The existence of transitive relationships underlies the presence of cohesively connected subgroups of children in classroom social networks (Orman et al., 2013; Santos and Vaughn, 2018), which have been shown to be predictive of children and adolescent developmental pathways (Daniel et al., 2015; Santos et al., 2015).

The great number of available social partners creates a complex social world that children have to navigate. To successfully do so, they need to accurately perceive available social information (Crick and Dodge, 1994). In school settings, interpersonal perceptions in general (i.e., one’s understanding of self and of others that results from social interactions) and accurate perceptions of classroom relationships in particular (i.e., knowing who is related to whom) facilitate children and adolescents’ academic and social functioning. Higher perception accuracy of classroom relationships is expected to give children the capacity to modulate their behavior to specific situations (e.g., which group to join for an activity) and help them navigate the classroom relational context (Cappella et al., 2012). For example, children with accurate perceptions of who likes and dislikes them tend to score lower on loneliness and internalizing behavior scales (Cillessen and Bellmore, 1999). Also, children with higher depressive symptoms tend to underestimate the number of friends they have, while aggressive children (who experience low levels of victimization) tend to overestimate this number (Morrow et al., 2016).

Recently, Daniel et al. (2017) showed that the perception accuracy of affiliative relationships in school children varies over time. But although there is correlational evidence suggesting that some individual-level variables (age, number of social connections) and classroom-levels variables (class size, emotional climate) predict perception accuracy of affiliative networks (Cappella et al., 2012; Neal et al., 2016; Daniel et al., 2017), no study has yet related changes in peer groups’ structure with changes in perception accuracy of social networks. Therefore, this study aims to contribute to the interpersonal perceptions’ literature by exploring this hypothesis directly and testing whether changes in an important structural feature of peer groups (transitivity) associate with changes in perception accuracy of affiliative networks.

To test this hypothesis we further analyzed the longitudinal data described in Daniel et al.’s (2017) study of perception accuracy of affiliative networks. Our prediction is that changes in transitivity will be positively associated with changes in perception accuracy of affiliative networks. This prediction is grounded on the fact that transitivity is a structural feature that indicates how much observed relationships depart from a random pattern of ties (Davis, 1970; Snijders et al., 2006). This way, we expect less randomness to lead to higher perception accuracy. Our prediction is also supported by recent experimental findings showing that recall accuracy of novel social networks is higher for social networks characterized by higher levels of transitivity (Brashears, 2013; Brashears and Quintane, 2015).

## Materials and Methods

### Participants

Participants of this study were children and adolescents from three longitudinal samples, from urban schools in the Lisbon metropolitan area (Portugal), originally described in Daniel et al. (2017). In the Portuguese school system, classrooms are self-contained groups of classmates that share the same schedule, space and teacher(s) throughout the year. To be included in this study participants had to complete the Social Cognitive Maps procedure in more than one time point and to belong to classrooms with participation rates >0.60 (mean classroom participation rates: Sample 1 = 0.83, Sample 2 = 0.77, and Sample 3 = 0.83). Participants provided assent and were given written informed consent from their parents to participate in this study.

#### Sample 1

145 7th graders (72 girls), observed once a year (spring) for three consecutive school years, from two public schools with a mean age of 12.49 years old (*SD* = 1.00) at the first data point. The number of classrooms which participants belonged to varied across years due to academic retentions and some reshuffling of classroom (year 1 = 12, year 2 = 18, and year 3 = 15).

#### Sample 2

40 2nd graders (19 girls), observed once a year (spring) for 3 consecutive school years, from one private school (3 classrooms) with a mean age of 7.19 years old (*SD* = 0.47) at the first data point.

#### Sample 3

72 elementary school children (48 girls), observed twice in a school year (February and June), from one public school (5 classrooms). No age data is available for this sample, although generally most 2nd, 3rd, and 4th graders in the Portuguese school system are 7, 8, and 9 years-old (respectively) in February.

### Procedure

Following the Social Cognitive Maps (SCMs) procedure (Cairns et al., 1985), participants were given a questionnaire and asked to list groups of classmates who hanged around together a lot and also to identify those who did not hang around with any particular group.

### Measures

#### Perception Accuracy

Because participants were asked to provide information of their own affiliative relationships and those of their peers, SCMs’ data can be used to compare participants’ perceptions of the affiliative network with the “true affiliative network.” We used Krackhardt’s (1987) locally aggregate structures intersection rule to identify the “true relationships” that compose classrooms’ affiliative networks. According to this rule a tie is considered to be present if and only if both parties in a dyad report the tie (i.e., a true relationship between A and B exists if both A and B report they hang around together). Consequently, only relational data of participants who completed SCMs were considered in this study.

To measure participants’ perception accuracy of affiliative relationships we used Brashears et al.’s (2016) accuracy index. This is index is computed by multiplying “precision” (i.e., number of correctly identified “true” relationships divided by the total number of relationships depicted in participant’s SCM) by “coverage” (i.e., number of correctly identified “true” relationships divided by the total number of “true” relationships). This index equals one when a participant’s SCM only depicts all “true” relationships. Lower index values occur whenever a participant’s SCM omits “true” relationships and/or includes relationships not identified as “true.” For example, imagine that a child depicted 35 relationships in her SCM. Suppose that 20 of these are in fact true relationships of the classroom network (15 incorrectly identified relationships), that comprises a total of 50 relationships (30 relationships omitted). In this case, precision equals 20/35 = 0.57, and coverage equals = 20/50 = 0.40. Consequently, accuracy equals = 0.57 × 0.40 = 0.23.

#### Transitivity

For each classroom “true” affiliative network we computed a transitivity index. This index ranges between 0 and 1, and captures the likelihood of one’s affiliates to also be affiliated with one another. It can be computed as follows: three times the number of transitive triads (participant i ↔ participant j, j ↔ k and k ↔ i), divided by three times the number of transitive triads plus the number of two-paths (i.e., triads which could become transitive with the addition of a single tie; i ↔ j and j ↔ k, but not k ↔ i).

### Data Analysis

We modeled changes in perception accuracy, for the three samples separately, using longitudinal random intercept models with repeated measures of perception accuracy nested within participants (i.e., participants – level 2, repeated measures – level 1). Models were fitted using lmerTest package in R version 3.4.2 (R Core Team, 2015; Kuznetsova et al., 2016) and included three predictors: Time, Transitivity at Time 0, and Transitivity change.

Time predictor represents the estimated change in perception accuracy scores between each time point, controlling for transitivity effects. Because transitivity is a time varying predictor it contains information about both between-participants and within-participant differences that requires disaggregation (Curran and Bauer, 2011). To disaggregate these differences we used Transitivity at Time 0 (first data point) to test between-participants’ differences, and computed participant-specific deviations from these values for each time point (Transitivity change) to test within-participants’ differences. This way, a positive effect (*β* > 0) of Transitivity at Time 0 indicates that participants in classrooms with higher transitivity at the first time point have higher perception accuracy of affiliative networks, while a positive effect of Transitivity change indicates that increases in transitivity associate with increases in perception accuracy.

## Results

Table 1 presents means and standard deviations for perception accuracy and transitivity scores across time and sample. Longitudinal random intercept models (Table 2) show that, for all samples, Transitivity at Time 0 significantly predicted perception accuracy of affiliative networks, with children and adolescents in classrooms with higher transitivity at the first time point showing higher perception accuracy (*β*_Sample1_ = 0.67, *β*_Sample2_ = 0.33, *β*_Sample3_ = 0.27; all *p’s* < 0.05). In more colloquial terms, and because transitivity and accuracy range between 0 and 1, an absolute 10% classroom difference in transitivity in Sample 1 at Time 0 (e.g., 0.50 compared to 0.40), is expect to lead to an absolute 6.7% difference in accuracy (3.3 and 2.7% for Samples 2 and 3, respectively).

**Table 1:** Descriptive statistics.

	Time 0	Time 1	Time 2
	
	*M (SD)*	*M (SD)*	*M (SD)*
**Perception accuracy**			
Sample 1	0.56 (0.21)	0.53 (0.21)	0.59 (0.18)
Sample 2	0.22 (0.15)	0.41 (0.16)	0.30 (0.13)
Sample 3	0.28 (0.22)	0.32 (0.20)	
**Transitivity**			
Sample 1	0.79 (0.16)	0.78 (0.17)	0.79 (0.12)
Sample 2	0.51 (0.35)	0.64 (0.13)	0.48 (0.14)
Sample 3	0.33 (0.31)	0.61 (0.21)	

**Table 2:** Transitivity effects on the perception accuracy of affiliative networks.

	Sample 1			Sample 2			Sample 3		
	*β*	*SE*	*p*	*β*	*SE*	*p*	*β*	*SE*	*p*
**Fixed effects**									
*β*_0_ Intercept	0.01	0.06	0.841	0.08	0.04	0.041	0.20	0.03	<0.001
*β*_1_ Time	0.01	0.01	0.318	0.05	0.02	0.004	-0.02	0.04	0.648
*β*_2_ Transitivity at time 0	0.67	0.07	<0.001	0.33	0.06	<0.001	0.27	0.07	<0.001
*β*_3_ Transitivity change	0.62	0.06	<0.001	0.40	0.06	<0.001	0.20	0.09	0.035
**Random effects**									
*σ^2^* Intercept	0.00			0.00			0.01		
*σ^2^* Residual	0.03			0.01			0.03		
**N/Observations**	145/357			40/117			72/144		

*Transitivity and accuracy indices range between 0 and 1.*

Additionally, changes in transitivity were positively associated with changes in perception accuracy of affiliative networks (*β*_Sample1_ = 0.62, *β*_Sample2_ = 0.40, *β*_Sample3_ = 0.20; all *p’s* < 0.05). Again for Sample 1, an absolute 10% increase in transitivity is expected to lead to an absolute increase of 6.2% in accuracy.

## Discussion

The goal of this study was to test whether changes in perception accuracy of affiliative relationships in elementary school children and young adolescents are related to an important structural feature of affiliative networks – transitivity (transitive relationships exist whenever one’s affiliates also affiliate with one another). As predicted, across all samples participants in classrooms with higher transitivity were better at perceiving who affiliated with whom, and increases in transitivity associated with increases in perception accuracy.

These findings provide support to previous suggestions that classroom-variables play a role in fostering accurate perceptions of social relationships (Cappella et al., 2012; Neal et al., 2016). Also, past findings show that involvement in transitive relationships associates with more favorable development outcomes, such as better social competence or higher number of friends (e.g., van den Oord et al., 2000; Daniel et al., 2015; Santos et al., 2015). Our results add to the pool of evidence showing associations between structural features of peer groups and individual adaptation.

Being transitivity a marker that indicates how much relationships depart from a random pattern of ties (i.e., how structured affiliative relationships are), these results suggest that consensual and accurate views of classroom social structure appear hand in hand with structural features that reflect more stable patterns of interaction. Structured and predictable social environments have been suggested to decrease the cost of social interactions, freeing individuals to explore additional social resources (Flack et al., 2013). The predictability of social relationships is expected to translate into an overall increase of social opportunities (more affiliative interactions and greater partner diversity) (Flack et al., 2006; Barrett et al., 2012). Understanding the interplay between network structure, perception accuracy and social interactions, and their joint contributions for social adjustment is thus an interesting avenue for further research with relevant implications in contexts where important social interactions take place, such as classrooms.

There is a growing body of evidence showing that teachers’ classroom management practices play an important role in shaping the pattern of peer relationships (Farmer et al., 2011). For instance, teachers who provide higher levels of emotional support to students have classrooms with higher rates of friendship reciprocity (Gest and Rodkin, 2011). As far as we know, there are no studies addressing the role of teachers’ “invisible hand” in other structural features of peer groups besides reciprocal relationships. It could be interesting to study whether teachers’ influence also extends itself to more complex structural features like transitive relationships. If this is the case, then, through the links between transitivity, perception accuracy of social relationships and social adjustment, teachers may indirectly benefit their students by creating classroom ecologies that foster transitivity. One suggested way to do so is to use seating arrangements and small group activities that join children who have common associates but that do not necessarily associate with one another.

Despite transitivity’s central role in peer group relationships there are other important network features that influence network dynamics (e.g., popularity, gender homophily) (e.g., Daniel et al., 2016). Future studies could explore how changes in other network features associate with perception accuracy changes as well. These data could potentially provide additional information about the relevance of different features for network dynamics.

## Ethics Statement

This study was carried out in accordance with the recommendations of APA Ethical Guidelines with written informed consent from all subjects. All subjects gave written informed consent in accordance with the Declaration of Helsinki. The protocol was approved by the Comissão Nacional de Proteção de Dados.

## Author Contributions

JD and AS designed the studies. AS partially collected the data and critically revised the manuscript. JD ran the data analysis. JD and RS interpreted the data and drafted the manuscript. JD, RS, and AS approved the final version to be published.

## Conflict of Interest Statement

The authors declare that the research was conducted in the absence of any commercial or financial relationships that could be construed as a potential conflict of interest.
